# Prevention of modern slavery within sex work: Study protocol of a mixed methods project looking at the role of adult services websites

**DOI:** 10.1371/journal.pone.0285829

**Published:** 2023-05-18

**Authors:** Rachel Keighley, Teela Sanders

**Affiliations:** School of Criminology, University of Leicester, Leicester, United Kingdom; University of Central Lancashire, UNITED KINGDOM

## Abstract

**Background:**

The core challenge this study addressed is that Adult Services Websites (ASWs) are an extensive and pervasive feature of the digital world that facilitate the advertising, negotiation and purchase of sexual services yet are also considered to be harbourers of sexual exploitation, modern slavery and human trafficking (MSHT) Giommoni L. et al. 2021, Milivojevic S. et al. 2020, Sanders, T., et al. 2018. Whilst awareness of cases of internet facilitated MSHT has entered the public and policy domain, little is known about the role and responsibilities of ASWs in this domain. Collaboratively with our partners, the findings from this study will first contribute to understanding how ASWs facilitate exploitation and second how they can become part of the prevention mechanisms and reporters of crimes.

**Methods:**

We present the design of our mixed methods study, underpinned by a peer Action Learning Set (ALS) approach. By working closely with ten survivors of sexual exploitation from 7 countries, the peer group have informed the study through participation in the advisory group, instrument design, implementation, analysis and dissemination. A training and support needs analysis prior to engagement in the research project ascertained what skills people would bring, what they would need for further personal and career development and if there were any additional requirements to enable participation. We provided capacity building through a bespoke training package over the project’s lifetime.

**Discussion:**

Conducting a peer-researcher ALS project informs the research topic by both empowering survivors of sexual exploitation, whilst engaging with their expertise and lived experience to shape the methods and focus. The summative evaluation of our methods informs wider peer research methodologies, little utilised in the field of MSHT research. Thus, this research produces evidence which acknowledges survivors as experts with value towards social science research.

## Background

Increasingly, sex work has moved online, and is now considered the largest sector of the UK sex industry with twelve distinct sex work-related online environments identified [1–3]. The Internet has played a crucial role in improving working practices for sex workers [4]. Furthermore, levels of violent crime experienced online are typically lower than those reported by sex workers offline [3]. However, among the positives of digitised sex worker and the use of adult service websites, the internet has afforded new opportunities for modern slavery and human trafficking [5].

Research suggests that digital technologies both facilitate and support traffickers to exploit sex workers [6–8]. Modern slavery is a complex, harmful and largely hidden crime [5]. In England and Wales, modern slavery is an umbrella term that covers different forms of exploitation including human trafficking, labour exploitation, criminal exploitation, sexual exploitation, and domestic servitude.

UK operational activity to tackle modern slavery, human trafficking and labour exploitation, is driven by law enforcement agencies, criminal justice partners across England and Wales, Scotland, and Northern Ireland [5]. Key partners include the Police, Crown Prosecution Service (CPS), the National Crime Agency (NCA) and equivalent agencies in Northern Ireland and Scotland. Crucially, the Home Office works closely with the Modern Slavery Policy and Evidence Centre (MS PEC), an independent research consortium. The MS PEC has brought together academics, policy makers, NGOs and businesses to generate knowledge, improve collaboration and strengthen the UK’s response to modern slavery based on evidence and research to inform policy and operational responses to modern slavery.

There are some significant legal and regulatory consequences of the sex industry moving online [3]. In March 2022 the UK Government published their latest draft of the Online Safety Bill [9], setting out some comprehensive legislative measures, underpinned by an understanding that the criminal law applies to online activity in the same way as to offline activity. An amendment to the Online Safety Bill targets online adverts for sexual services in an attempt to stop traffickers using online platforms to exploit people. Within scope of the Bill is harmful advertising content that advertises for illegal services, including adverts that facilitate MSHT [10]. In Section 52 and 53 Sexual Offences Act (2003) (Schedule 7 –Priority Offences) under *Sexual Exploitation* the Bill explicitly mentions “causing or inciting prostitution for gain” and “controlling prostitution for gain” [10] as offences to be regulated. Thus, ASWs will have a duty of care to prevent their sites from being used to advertise victims for sexual exploitation. Yet, controlling prostitution for gain is interpreted very widely in the criminal courts [11]. Within the criminal law ‘controlling’ is used to criminalise any activities where somebody associates with a sex worker [12]. This suggests that, while internet-enabled sex work has been largely out of the scope of policy developments and police priorities, the Bill’s focus on modern slavery and controlling of prostitution may lead to increased surveillance of online spaces [13].

In January 2023, further amendments were made to the Bill following discussions with the Honourable Members for Dover, South Holland and the Deepings, and Maidenhead [14]. To better tackle illegal immigration encouraged by organised crime groups, the government plans to add Section 2 of the Modern Slavery Act to the list of priority offences. Section 2 makes it an offence to arrange or facilitate the travel of another person, including through recruitment, with a view to their exploitation. Furthermore, the government proposes to add Section 24 of the Immigration Act 1971 to the priority offences list in Schedule 7. Although the offences in Section 24 cannot be carried out online, paragraph 33 of the Schedule states that priority illegal content includes the developing of offences relating to the offences that are listed. Therefore aiding, abetting, counselling, conspiring those offences by posting videos promoting illegal offences (such as the promotion of illegal channel crossings) classes as an offence that is committed online, thereby is included as priority illegal content. The result of this amendment would therefore mean that platforms must proactively remove that content.

Sexual exploitation is continually conflated with the broader sex industry landscape, such that labour exploitation and rights, are not addressed effectively [15]. Thus, there is a need to develop intelligence to understand the sex industry and its working practices online whilst also protecting people from exploitation. Future policy developments need to consider the scope of sexual services, understand the wider industry and which aspects of sex work are harmful or exploitative, rather than assuming all forms of sexual services are inherently harmful [3].

Knowledge of the structure and regulatory frameworks of ASWs and those that engage in online sex work is in its infancy and the UK has very little insight into the potential effects of greater regulation of ASWs [4, 16]. Thus, key to developing policies that protect sex workers from harm, is identifying key risk priorities that distinguishes between consensual sexual practices and exploitation and assessing the impact of policies and services to meet these priorities [1, 17]. The role of the Online Safety Bill should be to work collaboratively with ASWs and stakeholders in the sex industry to reduce vulnerability and criminality online, whilst maximising trust and confidence. Due to the multitude of risks and issues facing sex workers in various settings, a range of different types of support are required [1]. In-depth evidence has the potential to build a more accurate and comprehensive picture of the sex industry, that acknowledges its needs and the distinction between sex work as a consensual industry and human trafficking and abuse. It is the latter that needs to be prevented, whilst the former is regulated only to the extent that sex workers can operate freely and safely online. The Online Safety Bill must work collaboratively to encourage those in the sex industry to report crimes and abuse [15]. This approach will prioritise the safeguarding of those being harmed in the sex industry and will ensure the focus is on vulnerability and safety and a consistent approach across the online sphere.

## Methods

This mixed methods study employed interviews with police, first responders and ASWs and an online survey with sex buyers to generate a unique body of evidence that reaches beyond existing data or government consultation. Through this four-way approach to gathering data we mitigated against any risk of ASW operatives and professionals not co-operating. This provided a 360-degree perspective of ASW activity and enabled several strands of recruitment into the project maximising chances of sample size realisation.

### Ethical considerations

This study has been approved by the University of Leicester’s Research Ethics Board (approval ID #35229). Prior to interview data collection all participants were given a detailed participant information sheet, outlining the research aims, their role, and how we would collect, store and use their data. The researchers read out the consent form at the beginning of the interview and recorded the verbal consent of the participants. Consent is not assumed to be continuous, therefore participants had the option to withdraw without giving a reason and their data would be deleted up until 31^st^ January 2023.

For the survey, the participant information sheet and consent form was embedded within the survey tool. Agreeing to complete the survey was taken as consent of participation. Withdrawal was possible, up until the participant submitted the survey.

All research participants were safeguarded from emotional distress and a document containing relevant support services made available to them if they required further support.

### Data sources and tools

The core challenge to be addressed in this research was the fact that Adult Services Websites (ASWs) are an extensive and pervasive feature of the digital world across the globe that facilitate the advertising, negotiation and purchase of sexual services yet are also considered to be harbourers of sexual exploitation, modern slavery and human trafficking (MSHT) [3, 7, 18]. The policy and legal landscape under which regulating ASWs falls is significant, including the ongoing development of the UK Online Safety Bill [19]. To address gaps in the literature and practice knowledge this project had three core partners: National Crime Agency (NCA), National Police Chief’s Council (NPCC) and the leading NGO for the prevention of modern slavery in the UK, Unseen (who run the national helpline for victims).

Collaboratively with our partners this study first aimed to understand how ASWs facilitate exploitation and second how they can become part of the prevention mechanisms and reporters of crimes. The objectives centred on creating new knowledge and understanding to influence future law and policy reforms. Our objectives were fourfold:

1. To improve evidence on how Adult Services Website businesses can act as prevention stakeholders in MSHT by finding out what data they would need to keep to facilitate police investigations and good practice mechanisms around safeguarding;
2. Gather the views of ASWs operators and those who engage with buying sexual services regarding prospective laws to regulate ASWs in order to give insight into how regulatory reform should be designed to ensure compliance;
3. Identify the risks associated with policing around ASWs regarding victim identification and apprehending offenders to ensure any regulation does not displace MSHT or make spotting victims harder;
4. Establish a national network of stakeholders working with ASWs for long term action planning and consolidation of expertise to speak directly to law, policy, guidance and practice

Thus, our research questions centred around these objectives and informed the methods of study:

1. How can ASWs be used to disrupt offenders? What are the mechanisms adopted by ASWs for identifying victims and what do they do with the information?
2. How do operators of ASWs see their role in preventing abuses?
3. How do users of ASWs (providers and consumers) perceive their role in identifying crimes, barriers to reporting and prospective further criminalisation?
4. What are police expectations on the usefulness of tighter control of ASWs in victim interventions and apprehending offenders?

These research questions, alongside a multi-method approach offered a holistic view of the problems to be answered, and hopes to encourage collaborative working between ASWs, the police and policy makers tasked with preventing MSHT online.

### Peer researchers and Action Learning Set

A growing body of research and associated policies advocates for meaningful involvement of stakeholder communities, particularly those historically marginalised, in the design, development and administration of the research that affects them [20–22]. This project relied on Unseen for ethical survivor involvement as a peer research project. We worked under Unseen’s Survivor Involvement protocol and strategy to inform the setup of the project and the ethical approach to involving adult survivors of exploitation. Ten peers from seven countries were involved through participation in the advisory group, instrument design, implementation, analysis and the dissemination phase. All ten peers engaged throughout the project, however a protocol was in place such that they were free to withdraw at any time, at which point a further round of peers would be hired if any withdrew from the programme.

A training and support needs analysis was completed at the beginning of the project to ascertain what skills people would bring, what they would like to develop for further personal and career development and if there were any additional requirements they needed to enable participation (e.g. interpreting services, childcare costs, travel costs and/or payment). Consequently, provided capacity building through an Action Learning Set (ASL) methodology, made up of five sessions over the lifetime of the project, which was given to Unseen as a tool for peer research methods in MSHT research. The training needs analysis and input from the peers guided these sessions, which included research skills (survey and interview), researcher reflexivity, data analysis, writing for different audiences, impact and dissemination including creating resources.

An Action Learning (AL) approach acknowledges the agency and expertise of researchers as active participants and co-researchers [23]. Academic knowledge is often assumed to be derived from academic researchers. Action learning and peer research methodologies acknowledge and encourage peers with lived experience to create knowledge through their experiences [24]. Action learning is inclusive and understands the value of non-academics as experts with knowledge of the research topic [23]. Thus, research does not necessarily strive to be objective, but inclusive of lived and diverse experiences. This not only recognises the agency of co-researchers but also critiques the ‘objective’ academic researcher who assumes they know right from wrong.

Practicing an inclusive AL method for the purposes of this project, meant considering and integrating the beliefs, experiences and skills of survivors of MSHT as co-researchers within the study. Action learning meant: asking fresh questions; learning from and with one another; working collaboratively on solving complex research problems; sharing experiences, ideas, feelings; and critically reflecting on what works and what does not, how and how not, and why or why not [24]. Bawden and Williams [25] argued that this approach challenges current world views and the status quo, a necessary prerequisite for social science research whose whole purpose is to enact change and challenge previous knowledge or practices.

Successful peer research methods require training where peers lack experience which can add complexities and extra time commitments to a project [26]. Without adequate training in research skills, the validity and reliability of research findings can be questioned and undermined, thus limiting its impact. Lushey and Munro [26] argue that participatory peer research methods must address ethical, practical and data quality issues from the outset by providing adequate resources and effective research management to deliver and upskill participating peers. Peer research methodologies that adopt this approach avoid being tokenistic, in which the researchers are listened to, supported in sharing their views and ideas, are involved in decision making processes, and have some power and responsibility [27]. It is also important, however, to acknowledge the ‘insider’ and lived experience knowledge peer researchers bring to a project that can offer rich insight into the topic [26].

Thus, each session with the peer researchers began with a start-up skills block wherein co-researchers got to know the background and aims of the study, the research skills to be implemented in that session and ask questions regarding what was to be expected [23]. This was facilitated by Unseen, who offered continual support, training and meetings to the peers. This therefore helped to foster a more relaxed environment for relationship building. Moreover, as per good practice guidelines [23] the academic research team provided participants with all materials needed before each session, with references to recommended reading, literature, and websites so everyone would be prepared to contribute and get the most out of each session. Also, a planned schedule for each workshop was made ahead of time, underpinned by a training needs analysis undertaken by the peer researchers, and matched to the various stages of the research.

At each session, an overarching theme was chosen as the focus that both met the desires and interests of the peer researchers, whilst also matching the stage of the research project. The first two sessions were on data analysis (both qualitative and quantitative) whilst the next sessions focused on reflexivity, research impact and dissemination. During each session, the group critically reflected on their knowledge, and the purpose of each session, ending with a mutual, group-built understanding of the session’s objectives. Important to the academic researchers was a cascade of learning and knowledge to the peers, such that they had the confidence and the skills to meaningfully engage and share their insights and reflections in the research process. One aim of peer research methods is to encourage peer researchers to become competent researchers in their own rights, thus delivering skills training based on a training needs analysis was helpful in supporting this goal.

Upon completion of the Action Learning Set sessions, the peer researchers were awarded with a certificate of recognition, endorsed by the University of Leicester. This was to recognise their contribution to the research process as meaningful, as well as offering them a tangible document to add to their CV for future employment. The peer researchers were also given the opportunity to share their learning and findings in some public dissemination settings as well as contributing to journal outputs. The community events had the benefit of being a more relaxed environment for dissemination as well as raising awareness in the community regarding research findings to lead to positive change.

### Qualitative methodology → sample → procedures → analysis

We successfully conducted 51 interviews with the police (n = 30), practitioners (n = 13) and those working in the Adult Service Website industry (n = 6). The interview tools were jointly devised by the research team and peer researchers, with input from the partners who were used to assess the feasibility and focus of the research questions. Our engagement plans included utilising contacts through the NCA and police networks and snowball sampling as participants recommended others with the relevant subject matter experience. Relying heavily on the dynamics of organic and collaborate social networks it was important to design a strategic engagement plan prior to data collection to ensure maximum uptake. Our research partners proved instrumental in this process given their active involvement in this field.

In-depth interviews with ASW staff aimed to understand how ASWs viewed their role and procedures in identifying offenders of MSHT and their appetite for working alongside law enforcement in preventing sexual exploitation. The in-depth interviews with police and practitioners involved with ASWs engagement and enforcement aimed to understand their experiences of MSHT cases on ASWs and their expectations on the usefulness of tighter control of ASWs, including the feasibility and applicability of the Online Safety Bill. Together, these three interview groups offered a 360-degree insight into adult service website operations and the engagement of working parties in preventing MSHT. An omission of any one of these interview groups would overlook necessary and important information from groups that are and ought to be working together collaboratively to prevent exploitation online.

The interview questions were designed collaboratively with the peer research group, engaging both their expert knowledge of adult service websites, and specific areas of focus regarding police and platform responsibility in responding to MSHT in ways that centred the victims’ experiences free of judgement. An advantage of working with survivors in peer research is evident in achieving a holistic view of a research topic. Researchers, who whilst hold skills in emotive and sensitive research, lack the lived experiences offered by the peers, whose on the ground information about the realities of working online, experiencing exploitation and can attest to the needs of sex workers online can drive the research in a direction that respects sex workers’ rights to safety in ways that is meaningful for them. This research, as a peer research methodology, centred and prioritised sex workers’ experiences and desires, whilst also working collaboratively alongside law enforcement and policymakers to shape the laws to maximum effect.

The interviews were carried out and recorded via Microsoft Teams. Once the recording was completed the protocol, by default, meant the audio files were safely transferred to the designated Microsoft Teams research folder (a secure research file store used at the UoL), accessible only by the four interviewers, whilst the original recordings were deleted from the worker’s computer. This was to ensure maximum confidentiality and security. The audio files were then sent via a secure transfer to transcription service, Transcribe It, with a confidentiality agreement in place with the University of Leicester. This service provided verbatim Microsoft Word 2017 files which gave a transcription of the interviews. This was sufficient for discursive analysis of the interview data. The raw data was not shared across the team to protect the anonymity and confidentiality of research participants. The original recording will be destroyed five years after the project has ended, as is the University’s protocol.

All interview participants were informed of their rights and how their data would be collected, stored and used through a detailed participant information sheet shared prior to the interview. This has been approved by the University of Leicester’s rigorous ethics process. Consent was not assumed to be continuous, therefore participants had the option to withdraw without giving a reason and their data to be deleted up until 31^st^ January 2023.

The interview data was analysed using thematic analysis [28]. Thematic analysis was presented through thematic networks: ‘web-like illustrations (*networks*) that summarise the main *themes* constituting a piece of text’ [28: p.386], which enabled an emotionally sensitive, insightful and rich exploration of the data’s patterns. Thematic networks are an analysis tool which, similar to augmentation process, explores the connection between the explicit statements made by respondents and the implicit meanings, drawing on context [29]. They are a clear way of organising the data, through unearthing key themes using a representational tool that is accessible and easy to interpret [28].

The peer researchers assisted in identifying the key themes. Taking into consideration the inexperience of the peer researchers, training on thematic analysis was first delivered to offer a basic understanding of how to develop a codebook and identify key research themes from the findings. The peers’ lived experience proved useful in this regard, as they had an understanding of the types of themes likely to arise. The codebooks were then finalised by the academic research team to ensure academic robustness. It could be argued that this approach results in only partial participation in the analysis stage [26]. However, pragmatically, this was the correct decision, both due to the time limitations and commitments of the peer researchers on the project, and the experience of academics on data analysis tools. However, many of the themes adopted were both identified and named by the peers, rather than imposed by academic researchers during their analysis.

### Quantitative methodology → sample → measures → analysis

Using JISC’s Online Survey (certified to ISO 27001 standard), we carried out a survey for consumers using ASWs, to understand how they perceive their role in identifying crimes, barriers to reporting and prospective further criminalisation in relation to MSHT on ASWs.

The survey was jointly devised by the research team and peer researchers, with input from the partners and was used to assess the feasibility and focus of the research questions. This both recognised the expertise and experience of the peers, whilst also empowering them as research experts. Moreover, in addition, the peer researchers were offered research training specifically on designing a survey, and consequently, were nurtured in their academic research skills.

We collected data from 150 survey participants to gather a breadth of experiences and opinions to draw informed conclusions. Our engagement plans included advertising on adult service website platforms, and utilising our research partner networks, many of whom (including Unseen, the NCA and NPCC) work closely alongside adult service websites to improve their reporting and support mechanisms regarding abuse, MSHT and other illegal or harmful activities. This research project recognises that those ASWs which engaged and advertised the research are those who are active in support and preventing MSHT on their sites. However, to reach those consumers of sex on less regulated ASWs we hoped to either engage through snowball sampling and word of mouth.

The survey collected basic socio-demographic data from participants, whilst preserving anonymity. This was to understand the diversity of consumers of sex on adult service websites. To assess participants awareness of sexual exploitation, a mix of closed and open-ended questions were asked. The questionnaire’s inclusion of some open-ended free text questions allowed participants the opportunity to share their experiences and opinions in an unbiased way, thus giving agency to respondents. The survey questions were also designed with the peer research group who drew on their experiences and knowledge of ASW workings to inform and direct the survey’s focus.

The survey data was held on the Online Survey confidential secure site until the survey collection concluded and then the data was moved on to the Microsoft Teams research folder. No identifying features were collected within the survey data, however if anyone disclosed any potentially identifiable information, all efforts were made to anonymise this data and the raw data was again not shared with the wider research team.

The data was analysed using Statistical Packaging for the Social Sciences (SPSS), using a range of descriptive and crosstab analysis tools, as well as thematic analysis for the open-ended descriptive questions. This allowed for an in-depth exploration of consumers’ of sex experiences on ASWs to understand how they perceived their role in identifying MSHT crimes, barriers to reporting and prospective further criminalisation in relation to MSHT on ASWs. Thus, we sought to understand both their experiences of MSHT online, the current role of ASWs in providing a safe environment, and their views regarding tighter regulation on the purchase and selling of consensual sex, as distinct from trafficking offences to be regulated. Consequently, this research sought to understand how the users of ASWs perceive their role in this field.

Through this multi-way approach to gathering data and subsequent analysis we mitigated against any risk of ASW operatives and professionals not co-operating given the on the ground insight and lived experiences of consumers of sexual services on a wide variety of ASWs. This multi-method approach provided a 360-degree perspective of ASW activity and enabled several strands of insight into the project, maximising our understanding of the research questions.

The peer researchers assisted in the basic quantitative data analysis only. This was due to the skill levels required to analyse data using SPSS. However, their role proved instrumental in designing and presenting the quantitative data, in particular for the dissemination of our findings to wider audiences, including ASWs, and the providers and consumers of sexual services. In this way, the findings of our research reach beyond academic or political interest, but impact communities with lived experience, who can benefit from the research, including developing an understanding of how the Online Safety Bill may impact on their experiences on ASWs. The peers lived experience was harnessed to present the data creatively, in a way that was meaningful for them. Throughout the process of action learning, they also engaged in academic data analysis and dissemination to provide them with the necessary skills to develop as academic researchers and to be equitably involved in all stages of the research project [30]. Together, we worked on complex issues creating knowledge that could both contribute to the field of academic research and community knowledge that may achieve sustainable solutions to safe working environments online. However, this must be complemented by carefully crafted policy developments, as highlighted and shaped by our data analysis.

## Discussion

This project will provide much needed evidence on the best approaches to MSHT prevention on ASWs at a critically political time. It is not yet known what the impact of Section 52 and 53 Sexual Offences Act (2003) (Schedule 7 –Priority Offences) under *Sexual Exploitation* within the Online Safety Bill will be, nor the addition of Modern Slavery as a priority offence, and there are concerns among sex workers and adult service websites that sex work will be conflated with sexual exploitation, resulting in a blanket ban of sexual services being advertised online. Whilst there is useful impact related knowledge and parallels that can be drawn from the USA after the introduction of FOSTA-SESTA, the UK has very little insight into the potential effects of greater regulation of ASWs [4, 16].

Upon dissemination, this project will achieve better understanding of ASWs amongst law enforcers and victim intervention practitioners, the links to online harm and exploitation and the role of ASWs in protecting their customers/users. We also expect there to be wider understanding for those who use ASWs (providers and consumers of sexual services) around reporting processes for MSHT and sexual exploitation. Using the findings to influence the work of government groups should target harden the ASW industry against misuse by traffickers and disrupt MSHT offending, leading to swifter identification and more effective safeguarding of MSHT victims. This in turn will influence recommendations regarding the regulation of ASWs and sex work, policing guidance and operational practice via NPCC and future priorities taken by law enforcement agencies such as NCA.

By bringing together various groups who are already working in this space, new ideas and strategies that emerge between partners and ASWs can be taken forward into action plans for change to improve reporting, education and awareness as well as improving internal systems within ASWs to prevent exploitation. Thus, these findings will inform relationships with sex workers (including those at risk of, or who have been exploited), improving their safety and working environment online. Given the widespread concern by those working on ASWs that consensual working practices will be disrupted or banned, this outcome ought both to alleviate this worry, whilst simultaneously offering labour rights which respects workers’ rights to safety and freedom from exploitation.

This project extends knowledge gathered during the Government’s Online Safety consultation by addressing specifically the issue of ASWs, currently only implicitly addressed through government consultation. These findings will significantly enhance knowledge of online safety and harm. There is expectation that the Online Safety Bill will include provisions around preventing modern slavery and/or procuring prostitution but given this will not become law until 2024 this research can influence this agenda. Working collaboratively with the NCA, NPCC and close ties with the UK Home Office through the Modern Slavery Policy and Evidence Centre will ensure this new knowledge reaches these decision-making spaces. We plan to specifically ensure that these findings are directly integrated into the drafting of the law and any framework of governance around ASWs that is developed. The contribution by the peers in this process means we aim to inform the Bill in such a way that is meaningful to them, protecting the right to sell sex consensually online, whilst also protecting workers from exploitation and trafficking. Thus, beyond online harm, the findings will also be relevant to several areas of law and policy: notably future working groups associated with the Violence against Women and Girls Strategy; the Ten Years Drugs Strategy and other sex work governance.

Given the direct research implications on sexual services online, the findings will also be for evidence and action planning in several organisations working closely with the sex work sector. The NCA have a working group with ASWs working towards implementing a voluntary code of conduct. Our data will provide information to inform the development of this group and influence whistleblowing, moderation processes and enhance reporting of ‘red flags’. The APPG on Human Trafficking and Modern Slavery will be the main conduit for the findings so these can directly influence government policy making processes. The Human Trafficking Foundation will also be a key recipient of the findings as an umbrella organisation to influence all sectors and stakeholders preventing MSHT.

Unseen will develop a training package to flag how to spot harms, what ASWs do both in terms of good and bad practice, and how to effectively report. This training package will be for ASWs and for third party organisations about ASWs. Unseen will include ASW training in their training strategy going forward. They will create e-learning materials and videos with survivor guidance as key resources to embed in the training. To inform and update practitioners we will reach out to first responders, campaign and advocacy groups such as: Stop the Traffik; Survivors Alliance; Salvation Army; Medaille Trust; National Ugly Mugs, particularly in the analysis stage, recommendation development and dissemination. This will be done through online invited closed workshops as well as open dissemination events. Thus, the findings will contribute to knowledge and practice across the policy and working environments to ensure a cohesive and successful approach to preventing MSHT online is achieved.

Finally, one key objective is to facilitate the cross-fertilisation of knowledge between existing groups by forming a national stakeholder group which brings together people working with those affected by ASWs with those working in police and security intelligence. Currently, these are spread across policing, NGOs, security services and government departments but there is no overarching network. The strong partnerships and high levels of expertise in this area need co-ordination, a space for sharing knowledge and implementing change going forward. Such a stakeholder network will therefore become an established long-term route to influence ongoing policy and legal developments as well as service provision and interventions for those exploited. Unseen have offered to build this network as a result of this research project and the initial collaborations that will be generated.

The outcomes of this project will solidify the peer researcher approach for future research activities that aim to achieve real world impact, training, and knowledge transfer in the field of MSHT and wider research with vulnerable populations.

## Conclusion

This project will create new evidence, knowledge and understanding to inform issues on modern slavery in relation to ASWs and sex work governance, policing and regulation in the UK. We assess that in terms of academic contributions there will be interest from across disciplines who are working within a modern slavery framework concentrated in arts, humanities and social sciences as well as broader subjects such as informatics and computing. Criminology and criminal justice/policing studies as well as law, socio-legal studies and gender studies will be central areas where these findings will be transformative. The gaps in literature that will be filled relate to a) sex customers’ views on the regulation of ASWs and any models of criminalisation, regulation and governance; b) law enforcers’ view on what best ways to regulate ASWs and make businesses responsible; c) evidence on how ASWs can facilitate prevention through new and enhanced mechanisms of safeguarding processes. These findings will explicitly translate into practical applied knowledge. The new data will inform ongoing public debates around the governance of the sex industry in the UK and the interrelationship with modern slavery and effective safeguarding of victims.

## Supporting information

S1 File (PDF)

S2 File (DOCX)
