# Supplementary material for: Prevention of modern slavery within sex work: Study protocol of a mixed methods project looking at the role of adult services websites
Source: PLoS One. 2023 May 18;18(5):e0285829. doi: 10.1371/journal.pone.0285829 (PMC10194949; doi:10.1371/journal.pone.0285829)
Supplement: S1 File — (PDF) [file pone.0285829.s001.pdf]

Regulation Of Online Websites and The Prevention Of Sexual Exploitation

Page 1: Participant information sheet

This study seeks to understand how and in what ways Adult Services Websites (ASWs) can (knowingly or unknowingly) facilitate sexual exploitation, modern slavery and human trafficking (MSHT) abuses towards vulnerable people. The overall aim of the research is to engage with this important crime prevention issue.

The project is funded by the Arts and Humanities Research Council (AHRC) and the Modern Slavery Policy and Evidence Centre. More information on the project can be found here. The PI for the project is Professor Teela Sanders at the University of Leicester.

This research project will use an online survey and interviews to understand how ASWs can facilitate exploitation and how they and the people who use them can become part of the prevention and safeguarding mechanisms and reporters of crimes.

This survey is for Adult Service website consumers living and/or working in the UK who use the Internet to purchase sexual services. It asks about:

- your opinions on adult service websites,
- your experiences identifying exploitation and harms,
- barriers to reporting and,
- your view on the criminalisation or regulation of ASWs

The results of this research will be used to inform policy and practice in order to improve safeguarding and regulatory conditions for sex workers and purchasers on adult service websites so they are safe spaces for all.

Your contribution to this research is most welcome. Your results are anonymous and no identifying features are collected. The survey should take no more than 10-15 minutes to complete. Please complete the survey in one sitting, as if you leave it you will not be able to save your answers and resume the survey at another time.

If you have any questions about the survey, or the wider project, please contact Rachel Keighley by email at rek17@leicester.ac.uk

Page 2: Informed consent

For the purposes of this research:

An Adult Service Website is any website in which sexual services can be advertised and/or purchased.

Modern slavery encompasses human trafficking and slavery, servitude and forced or compulsory labour. In human trafficking cases, exploitation can take many forms, including: sexual exploitation, forced labour, slavery, servitude, forced criminality and removal of organs.

Sexual exploitation is the actual or attempted abuse of a position of vulnerability, power, or trust, for sexual purposes, including, but not limited to, profiting monetarily, socially or politically from the sexual exploitation of another.

Do you use Adult Service Websites to purchase sexual services? ✱ *Required*

- (•) Yes
- ( ) No

I agree to the following: ✱ *Required*

Please select exactly 4 answer(s).

- [ ] I confirm that I have read and understand the participant information sheet for this study
- [ ] I understand that my participation is voluntary
- [ ] I agree to take part in this research project
- [ ] I agree to the use of anonymised quotes in publications

Page 3: Introductory questions

Are you based in the UK? ✱ *Required*

- Yes
- No

How would you describe your gender? ✱ *Required*

- Male
- Female
- Non-binary
- Prefer not to say
- Other

If you selected Other, please specify:

What is your age group? ✱ *Required*

- 18-24
- 25-34
- 35-44
- 45-54
- 55-64
- 65 and over

How long have you been using Adult Service Websites to search for sexual services? ✱ *Required*

- Less than 1 year
- 1-2 years
- 3-5 years
- 6-10 years
- More than 10 years

Who do you normally buy sexual services from? ✱ *Required*

- Men
- Women
- Transgender men
- Transgender women
- Couples
- Prefer not to say

Page 4: Opinions on Adult Service Websites

When purchasing sexual services, where do you think the **majority** of the money goes? ✱ *Required*

Please select no more than 1 answer(s).

- ☐ To the sex worker
- ☐ To a third party (e.g. an agency)
- ☐ To the ASW
- ☐ Not sure / don't know
- ☐ Other

If you selected Other, please specify:

Page 5: Experiences identifying exploitation

When you have purchased sexual services on Adult Service Websites, have you ever experienced an encounter where you feel someone is being sexually exploited by another person? ✱ *Required*

- ○ Yes
- ○ No
- ○ Not sure

If yes, can you briefly describe the scenario(s)?

On a scale of 0-10, how far do you think it’s your responsibility to ensure you’re not purchasing sex from someone who is being sexually exploited by another person? ✱ *Required*

Please don't select more than 1 answer(s) per row.

Please select at least 1 answer(s).

	0	1	2	3	4	5	6	7	8	9	10	
Not my responsibility	☐	☐	☐	☐	☐	☐	☐	☐	☐	☐	☐	100% my responsibility

Do you perform any verification checks before making a booking? ✱ *Required*

- ☐ Yes
- ☐ No
- ☐ Not sure

When viewing adverts on Adult Service Websites, what are your **5 most significant** concerns that someone is being sexually exploited by another person? ✱ *Required*

Please select between 1 and 5 answers.

- ☐ Booking not made directly with the sex worker
- ☐ Age explicitly under 18 or implied (e.g. references to youth: ‘young and cute’ / ‘sweet’ / ‘fresh’ / ‘candy’)
- ☐ Sex worker’s weight/size <110lb/50kg
- ☐ Sex worker’s nationality/ethnicity
- ☐ Multiple sex workers in one photo
- ☐ Low quality photos
- ☐ Stock image photos
- ☐ Background of the photos look like non-private residence (hotel, etc.)
- ☐ Background of the photos looks rough/dodgy
- ☐ Neutral expression or sex worker showing distress
- ☐ Same contact number in different ads
- ☐ Same/similar text as other ads for different sex workers
- ☐ References to being new: ‘new in town’ / ‘just arrived’
- ☐ Poor grammar and misspellings in the text

- Use of emojis e.g.: peach/cherries/snowflake
- Little or no further information in Q&A section
- Long list of services offered in ad e.g.: 10 or more services
- Specific ‘risky’ services: bareback / anal / OWO (oral without a condom)
- Services offered at a low price
- Availability in multiple locations
- Low price
- Sex worker is deaf/disabled
- Other

If you selected Other, please specify:

When purchasing sexual services on Adult Service Websites what are your **5 most significant** concerns that someone is being sexually exploited when **interacting with the person**? ✱ *Required*

Please select between 1 and 5 answers.

- Evidence of substance misuse
- Evidence of harm (e.g. cuts or bruises)
- Possible eating disorder / appears very thin
- Looking ‘spaced out’
- Complained of feeling ill
- Looked under 18
- Appeared / looked scared
- Appeared naïve about providing sexual services
- Bait and Switch (not the person in the photo you booked)
- Can’t speak any English / limited English
- Third parties present at the venue
- Confinement/restricted movement
- Third party financial control
- Isolation
- Monitoring of sex worker by third party
- Sex worker is driven to/from their appointment
- Debt bondage
- Withholding documents
- Other

If you selected Other, please specify:

Either during the booking process or during an appointment what would you do if you suspected a sex worker was being exploited by others? ✱ *Required*

- Cancel the booking
- Report to the ASW
- Report to the police

- ☐ Continue to make the booking
- ☐ Write a review expressing concern
- ☐ Warn others who purchase sexual services
- ☐ Discuss with the sex worker
- ☐ Other

If you selected Other, please specify:

Page 6: Barriers to reporting

Are there reporting routes on the Adult Service Website (ASW) platform for you to report concerns in relation to sexual exploitation? ✱ *Required*

- Yes
- No
- Not sure

Have you ever reported something to the ASW platform? ✱ *Required*

- Yes
- No

Can you explain why you did, or did not report a concern to the ASW? ✱ *Required*

Did the ASW platform respond? ✱ *Required*

- Yes
- No
- Not applicable

If yes, are you aware of any interventions they took?

- Removed advert
- Monitored the advert
- Contacted the police
- Contacted a support service
- Contact NGO to report exploitation
- Contact the sex worker using the phone number on the advert / account
- Initiated a safeguarding visit with first responder
- No intervention
- Other

If you selected Other, please specify:

Have you ever reported a suspicious activity in relation to the purchasing of sexual services to the police? ✱ *Required*

- Yes
- No

Did the police reply to you? ✱ *Required*

- Yes
- No
- Not applicable

If yes, do you know the outcome?

What, in your experience, are the **2 most** significant barriers to reporting? ✱ *Required*

Please select between 1 and 2 answers.

- Don’t know where to report
- I wish to remain anonymous
- I don’t think any action will be taken
- I don’t trust the police
- It’s not my responsibility
- Other

If you selected Other, please specify:

Which of the following organisations would you trust to provide you with information about the risks of sexual exploitation evident in some parts of the sex industry? ✱ *Required*

- NGOs
- Police
- Adult Service Website
- UK Government
- NHS or healthcare provider
- Peer forums
- Other

If you selected Other, please specify:

Page 7: Regulation of Adult Service Websites

Should Adult Service Websites be more strictly regulated in relation to modern slavery, human trafficking and sexual exploitation? ✱ *Required*

- ◌ Yes
- ◌ No
- ◌ Not sure

Please give a reason for your answer ✱ *Required*

If yes, how should they be regulated to reduce the risks of exploitation and modern slavery and to prevent harms? ✱ *Required*

- ☐ ID checks/age verification for sex buyer
- ☐ ID checks/age verification for sex worker
- ☐ ASW monitors abusive language and hate
- ☐ Stricter and monitored payment processes
- ☐ Checks for independence of sex worker
- ☐ Other

If you selected Other, please specify:

On a scale of 0-10, to what extent should ASWs be responsible for monitoring and acting to deal with inappropriate material on their websites? ✱ *Required*

Please don't select more than 1 answer(s) per row.

Please select at least 1 answer(s).

	0	1	2	3	4	5	6	7	8	9	10	
No responsibility	☐	☐	☐	☐	☐	☐	☐	☐	☐	☐	☐	100% responsibility

Are you aware of the Online Safety Bill and the inclusion of inciting prostitution for gain? ✱ *Required*

- ◌ Yes
- ◌ No
- ◌ Not sure

The Online Safety Bill hopes to make the UK the safest place in the world to be online while defending free expression.

The Online Safety Bill imposes obligations on services regarding three types of content: **illegal content, content that is harmful to children, and content that is harmful to adults**. It then applies further sub-categorisations within these content types.

One sub-category is 'inciting prostitution for gain'. The UK law defines inciting prostitution for gain as:

1. Someone who intentionally causes or incites another person to become a prostitute in any part of the world, and
2. They do so for or in the expectation of gain for themselves or a third party

Thus, Adult Service Websites may be monitored and required to regulate against harms, including modern slavery, human trafficking and sexual exploitation.

Do you think a law such as the Online Safety Bill will have an impact on your choice to purchase sexual services? ✱ *Required*

- ○ Yes
- ○ No
- ○ Not sure

On a scale of 0-10, what effects do you think the Online Safety Bill will have on the sexual services marketplace more widely? ✱ *Required*

Please don't select more than 1 answer(s) per row.

Please select at least 1 answer(s).

	0	1	2	3	4	5	6	7	8	9	10	
No effect	☐	☐	☐	☐	☐	☐	☐	☐	☐	☐	☐	The shut down of Adult Service Websites

On a scale of 0-10, do you think the Online Safety Bill will have an effect on the safety of sex workers advertising services online? ✱ *Required*

Please don't select more than 1 answer(s) per row.

Please select at least 1 answer(s).

	0	1	2	3	4	5	6	7	8	9	10	
Very unsafe	☐	☐	☐	☐	☐	☐	☐	☐	☐	☐	☐	Much safer

What would you do if the Adult Service Website (ASW) that you normally use started asking you for more personal information or requiring a log in (in order to prevent or reduce harms associated with exploitation and modern slavery)? ✱ *Required*

- ☐ Continue to use the platform
- ☐ Move to a smaller/lesser known ASW
- ☐ Start purchasing services offline
- ☐ Purchase services on the dark web
- ☐ Stop purchasing services altogether
- ☐ Other

If you selected Other, please specify:

If greater regulation around sexual exploitation comes in, the ASWs may have to ask you for more personal information. What information would you be comfortable to share and have stored by the ASW? ✱ *Required*

- ☐ Email
- ☐ Bank details
- ☐ Name
- ☐ Contact number
- ☐ Home address
- ☐ Copy of ID
- ☐ Disclosure and Barring Service (DBS) check
- ☐ None of the above
- ☐ Other

If you selected Other, please specify:

If ASWS are more regulated, do you think it will discourage purchasers and providers from using visible/legitimate websites? ✱ *Required*

- ○ Yes
- ○ No
- ○ Not sure

On a scale of 0-10, to what extent do you think greater scrutiny of ASWs will make platforms less attractive to sex providers? ✱ *Required*

Please don't select more than 1 answer(s) per row.

Please select at least 1 answer(s).

	0	1	2	3	4	5	6	7	8	9	10	
No effect	☐	☐	☐	☐	☐	☐	☐	☐	☐	☐	☐	Discourage use of ASWs

Do you have any other comments on the regulation of Adult Service Websites?

Page 8: Thank you

Thank you for taking the time to fill out this survey. It is so important that the experiences of those in the sex industry are heard and safeguarded in law and policy making about sex work. We hope that the results of our research will help influence policy makers in the UK.

If you wish to report a suspected crime, or would like some information on finding assistance for interventions such as mental health support, sexual health services or support for transition out of sex work please contact Unseen at https://www.unseenuk.org/ or phone their Modern Slavery Helpline **08000 121 700** or report concerns online at https://www.modernslaveryhelpline.org/report

Thank you again for taking the time to complete the survey.
