# Supplementary material for: Prevention of modern slavery within sex work: Study protocol of a mixed methods project looking at the role of adult services websites
Source: PLoS One. 2023 May 18;18(5):e0285829. doi: 10.1371/journal.pone.0285829 (PMC10194949; doi:10.1371/journal.pone.0285829)
Supplement: S2 File — (DOCX) [file pone.0285829.s002.docx]

## Interviews for FIRST RESPONDERS

**OPENING QUESTION**

1. Tell us about your service and your specific role?

**HOW ARE FIRST RESPONDERS INVOLVED WITH ASWs**

1. To what extent does your work have a focus on supporting those who have experienced sexual exploitation (MSHT)?
   1. How have you supported victims of sexual exploitation?
   2. Could you tell me more about the characteristics of those you have supported? E.g. gender, nationality, immigration status etc.?
2. In what ways have individuals engaged on ASWs been victims of modern slavery and how much have you seen this?
3. How have you come to know about victims of crime who are engaged (voluntarily or coerced) through ASWs?
4. How effective are current processes in responding to MSHT on ASWs?
5. Do you think ASWs should be involved in the prevention of MSHT?
   1. What would work well and why?
6. Are you aware of the Online Safety Bill and its plans to make social media platforms more accountable for ‘harms’ including sexual exploitation?
   1. If so, do you think this will assist?
   2. What are the potential issues/challenges with this legislation?

## Interviews for adult service website operators

**OPENING QUESTIONs**

1. Can you tell me about your platform, how it works, where it is based and who your target audience is?
   1. What is your sign-up process?
2. How has your ASW approached the prevention of harms?
   1. Are you aware of how your approach fits into the wider landscape of what other ASWs are doing?
   2. What has changed with your approach over time and why?

**KNOWLEDGE OF MSHT**

1. How significant do you believe the issue of commercial sexual exploitation via ASWs to be?
2. What are your experiences of your platform being used to harbour sexual exploitation?
   1. To what extent do you believe that online platforms enable this and in what ways?
3. What do you believe the indicators of exploitation may be that you/your company would be able to see?
   1. To what extent and how do you proactively search for these?
   2. Do you think your approach is effective?

**EXPERIENCES WITH CASES OF MSHT**

1. Can you share a stats/qual case study example of how your processes have been successful in detecting modern slavery/trafficking cases?
2. What is your current policy on the prevention of modern slavery and human trafficking?
3. What is your platform doing to prevent traffickers from using your site?
   1. Do you believe ASWs should be responsible for preventing the occurrence of MSHT?
   2. Have you been involved in the voluntary gold standard code of practice with the NCA? What value has this got?
4. Are there reporting routes on the platform for the public to report concerns?
   1. Is this anonymous?
   2. What is the volume and nature of advertisers’ complaints and consumer complaints? Is any of it passed to LE?
   3. If so, how are these investigated?
   4. Is there a criterion?
5. Does moderation or use of algorithms take place on the platform to continue to detect issues and how does this work?
   1. How reliable is this?
   2. What barriers exist that may prevent comprehensive moderation processes?
   3. Are you planning to expand this in light of the OSB?
6. What further data and/or support do you need to appropriately respond to MSHT?

**WORKING WITH THE POLICE AND LAW ENFORCEMENT**

1. What have you done when you have been made aware of a potential case of sexual exploitation?
   1. What would be your process to take action? E.g., the practical process with escalating to law enforcement?
   2. What measures do you have to safeguard the victim/performers?
   3. What is your strategy for minimising the risks?
   4. How effective do you think this approach is? What could work better?
2. How have you engaged / co-operated with law enforcement in these concerns?
   1. What is the extent to which there is trust for sharing information with law enforcement?
   2. What barriers exist that may prevent you from proactively reporting welfare concerns to law enforcement, or supporting law enforcement requests for information?
3. Is there is sufficient information and feedback shared between yourselves and the police?
   1. For example, feedback from law enforcement to ASWs on the quality of referrals ASWs make where there are concerns around offending activity

**ONLINE SAFETY BILL AND REGULATION**

1. How might the inclusion of inciting prostitution clause in the OSB affect your business?
2. If you are required to add more verification process, how would this affect your business/platform?
3. Do you think there are ways that customers could be made more responsible i.e. de-anonymised use to verify the users?
4. What do you think the role of Ofcom could be in supporting online safety regulation?
5. Do you think there are other strategies that ASWs can introduce to assist with prevention?
6. What benefits or challenges do you think further laws/amendments could present and how might this affect your business? E.g., FOSTA-SESTA
7. What do you think the main challenges will be in the future for ASWs in relation to crime prevention?
8. What would ASWs find helpful from a policy perspective?

## Interviews for POLICE AND LAW ENFORCEMENT

**Can you explain your job role?**

**OPENING QUESTION**

1. What do you consider to be the current key issues with how ASWs are (un)regulated?

**HOW ARE THE POLICE INVOLVED WITH ASWs**

1. What are the core policing activities that you are involved with around ASWs and MSHT offences?
   1. What percentage of your operations involve these types of cases?
2. Have you been involved in any operations of MSHT on ASWs? – detail/outcome etc.
   1. How effective are current processes for policing MSHT on ASWs? How much did they disrupt activity?
   2. Are there concerns about the cost implications of these lengthy investigations?
   3. Were there any barriers to the investigations?
   4. Organised crime or smaller operations? International?
   5. What types of outcomes have been achieved in terms of prosecution?
3. Are you aware of any reporting routes on ASWs for the public to report concerns?
   1. Is there a preferred reporting route for victims or observers of MSHT?
   2. How effective do you think these reporting routes are?
   3. What other tools do the police need to investigate potential crimes?
4. Can you tell me about the types of partnership you have with services offering support to victims?
   1. How effective are these partnerships / National Referral Mechanism?
   2. What are the issues/challenges you face?
5. In your opinion, does the relationship between police and victims of MSHT on ASWs need to be improved to promote identification of victims, promote reporting and for protection of victims?
   1. Why/Why not?

**FOR SENIOR POLICE**

1. Do you know anything about the Home Office voluntary code of practice developed with ASWs and the NCA?
   1. How was this developed?
   2. What shape it is in?
   3. Does the code of practice work to identify who is vulnerable to MSHT?
   4. Who do you work with? E.g. creators, users of websites
   5. How successful do you think it will be and why?
   6. Do you think it has already had traction and in what ways?

**WHAT ARE ASWs DOING TO PREVENT CRIME**

1. What are you aware of that ASWs are doing to prevent MSHT being facilitated through their platforms?
   1. Are these successful strategies?
   2. Are there any issues with existing data ASWs provide with respect to MSHT?
2. Do you think there is more ASWs could do? If so, what kind of data would be useful for police investigations?
   1. How would this assist in prevention?
   2. Do you think ASWs have the capacity to influence practices in the sex work industry?
3. What do you think are the core barriers to tackling MSHT offending via ASWs?
   1. What are the barriers to victimless prosecutions?
4. How does the current law against sex work (e.g. brothels etc.) facilitate or introduce barriers to protect and prosecute?
   1. What is working and what is not with respect to legal powers and safeguarding vulnerable people?
5. Is there sufficient information and feedback shared between yourselves and ASWs?
   1. For example, feedback from law enforcement to ASWs on the quality of referrals ASWs make where there are concerns around offending activity
   2. What kinds of information do they share? Is it sufficient for you to act?

What can be done to make platforms less attractive to criminals?

**ONLINE SAFETY BILL**

1. What do you think the implications will be of the Online Safety Bill in terms of ensuring platforms are more responsible?
2. Will the OSB support the police in detecting MSHT on ASWs and in what ways?
   1. What issues might it create and what could be done about these?
3. What do you think is needed in terms of enabling Ofcom as the regulator to understand this terrain?
4. How do you think Ofcom should interact with the police in carrying out their investigations for compliance?
5. Do you expect any unintended implications of the OSB? E.g. displacement of activity?
6. What are your views on whether new regulation will push exploitation to margins (two tier – ASWs that are regulated vs unregulated sites)?

OR

1. What do you think the police should be doing to protect against MSHT on ASWs?
2. How can victims of MSHT on ASWs be empowered to report incidents to the police?
3. How can the law be shaped to prevent MSHT on ASWs?
4. Would you find training on policing MSHT on ASWs useful?
